# Lack of Association between Y-Chromosomal Haplogroups and Prostate Cancer in the Korean Population

**DOI:** 10.1371/journal.pone.0000172

**Published:** 2007-01-24

**Authors:** Wook Kim, Tag-Keun Yoo, Sung-Joo Kim, Dong-Jik Shin, Chris Tyler-Smith, Han-Jun Jin, Kyoung-Don Kwak, Eun-Tak Kim, Yoon-Sun Bae

**Affiliations:** 1 Department of Biological Sciences, Dankook University, Cheonan, Korea; 2 Department of Urology, Eulji Medical Center, Eulji University School of Medicine, Seoul, Korea; 3 Research Institute of Molecular Genetics, Catholic Research Institutes of Medical Sciences, Seoul, Korea; 4 Cardiovascular Genome Center, Yonsei University College of Medicine, Seoul, Korea; 5 The Wellcome Trust Sanger Institute, Wellcome Trust Genome Campus, Hinxton, Cambridge, United Kindgom; 6 Department of Urology, Eulji University School of Medicine, Daejeon, Korea; Ordway Research Institute, Inc., United States of America

## Abstract

The Y chromosome has recently been suggested to have an association with prostate cancer risk in human populations. Since this chromosome is haploid and lacks recombination over most of its length, haplotypes constructed from binary markers throughout the chromosome can be used for association studies. To assess the possible Y-chromosomal contribution to prostate cancer risk, we have therefore analyzed 14 Y-chromosomal binary markers in 106 prostate cancer cases and 110 controls from the Korean population. In contrast to previous findings in the Japanese population, no statistically significant difference in the distribution of Y-chromosomal haplogroup frequencies was observed between the case and control groups of Koreans. Thus, our data imply that the previously reported associations between Y-chromosomal lineages and a predisposition to, or protection against, prostate cancer might be explained by statistical fluctuations, or by genetic effects that are seen only in some environments.

## Introduction

Prostate cancer is one of the most common male-specific cancers, but its incidence varies considerably between populations, with the chance of developing this cancer being highest in Western countries and lowest in Asian countries. Recent surveys suggest that both genetic alterations and dietary factors may be linked to prostate cancer [1]–[5], although the etiology of this disease remains unclear in the majority of cases.

There is increasing evidence for a Y-chromosomal role in malignancy and male-specific cancer progression. Y-chromosomal mutations are associated with prostate cancer, since the loss of this chromosome is the most common chromosomal aberration observed in prostate cancer tissue [1], [6]. Many genes or loci on the Y chromosome may contribute not only to male germ cell development and maintenance, but also to the molecular mechanisms of development and progression of prostate cancer [7]–[10]. For instance, *SRY*, the sex determining gene on the Y chromosome, is down-regulated in this cancer and is a negative regulator of the androgen receptor [11]. The *SRY* gene thus seems to be candidate for involvement in the oncogenesis of prostate cancer [12].

The Y chromosome has special genetic features that include an absence of recombination over most of its length and haploid status. The DNA sequence of the non-recombining region of the Y chromosome therefore contains a record only of the mutational events that occurred in the past. As a consequence, haplotypes constructed from Y-chromosomal alleles have been successfully used to study paternal lineages [13]–[16] and to differentiate human population groups [17]–[20]. In addition, any mutation predisposing to, or protecting against, prostate cancer will lie on the well-established phylogeny, so that the binary markers that define the lineages can also be used for association studies. In addition, since Y-chromosomal lineages (i.e. haplogroups) are highly stratified among human populations, such a haplogroup-specific association is likely to be population-specific as well.

Interestingly, recent studies have suggested that certain Y-chromosomal lineages were associated with prostate cancer risk in the Japanese population [12], [21]. Such findings need to be replicated in an independent population sample where the relevant lineages are common. Based on the results of previous population studies, the Japanese appear to have a closer genetic relationship to Koreans than to other Asian populations [20], [22], [23] so the Korean population is particularly suitable to test for the same correlation.

In the present study, we have therefore investigated the association between Y-chromosomal haplogroups and a predisposition to prostate cancer in the Korean population by examining 106 prostate cancer cases and 110 controls using 14 Y-chromosomal binary markers.

## Results and Discussion

**Figure 1:**
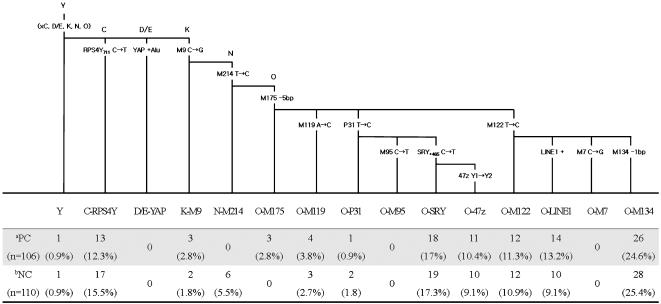
Y-chromosomal haplogroup distribution in prostate cancer cases and controls in the Korean population. The parsimonious tree on the top shows the evolutionary relationship of fifteen haplogroups. Nomenclature is according to the Y Chromosome Consortium [37]. ^a^Prostate cancer; ^b^Normal control; Exact *P* value = 0.44225±0.02442

We observed eleven different Y-chromosomal lineages defined by the fourteen binary markers in the cancer cases and control samples, most of which are the expected predominant haplogroups in east Asia. Frequency distributions of the fourteen binary markers and corresponding Y-chromosomal haplogroups are listed in Figure 1. The Korean population surveyed here is characterized by a high frequency of haplogroup O-M175 (and its sublineages) in both groups of prostate cancer patients (84.0%) and normal controls (76.3%) (Figure 1 and Table 1). This result is consistent with previous reports, showing that most of the east Asian populations share a common genetic feature of high frequencies of haplogroup O-M175-derived chromosomes [20], [24], [25]. The distribution of Y chromosome frequencies studied here was also concordant with previous results from Korean surveys [20], [25].

**Table 1:**
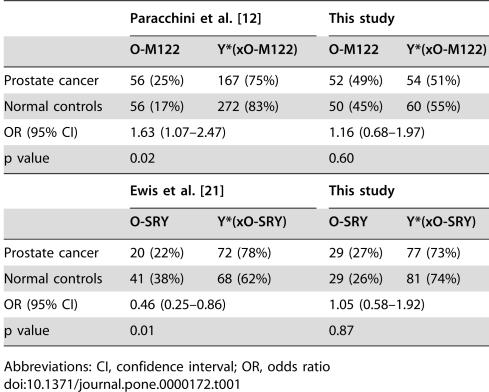
Association between prostate cancer risk and the Y-chromosomal haplogroups O-M122 and O-SRY in previous studies of the Japanese population, and in the Korean population

	Paracchini et al. [12]		This study	
	O-M122	Y*(xO-M122)	O-M122	Y*(xO-M122)
Prostate cancer	56 (25%)	167 (75%)	52 (49%)	54 (51%)
Normal controls	56 (17%)	272 (83%)	50 (45%)	60 (55%)
OR (95% CI)	1.63 (1.07–2.47)		1.16 (0.68–1.97)	
p value	0.02		0.60	
	**Ewis et al. [21]**		**This study**	
	**O-SRY**	Y*(xO-SRY)	**O-SRY**	Y*(xO-SRY)
Prostate cancer	20 (22%)	72 (78%)	29 (27%)	77 (73%)
Normal controls	41 (38%)	68 (62%)	29 (26%)	81 (74%)
OR (95% CI)	0.46 (0.25–0.86)		1.05 (0.58–1.92)	
p value	0.01		0.87	

Abbreviations: CI, confidence interval; OR, odds ratio

No statistically significant difference (p<0.05) in the distribution of Y-chromosomal haplogroup frequencies was observed between the case and control groups (Figure 1). We specifically re-investigated the previously-reported associations found in the Japanese population in the Korean samples. Paracchini et al. [12] reported that haplogroup O-M122-derived lineages (O3 in their paper) were associated with a statistically significant predisposition to prostate cancer in their Japanese sample. We did not find any significant association with prostate cancer risk in our samples of haplogroup O-M122-derived lineages (OR 1.16 (0.68–1.97), p = 0.60; Table 1 [Table pone-0000172-t002]), even though these lineages are more frequent in the Korean population than in the Japanese [12], [20], [25]. Neither stratifying by age (<65) nor by disease severity (using the criteria of Paracchini et al. [12]) led to a significant association (OR 1.50 (0.64–3.50), p = 0.35; OR 1.09 (0.59–2.02), p = 0.77, respectively; Table 2). Ewis et al. [21] found that haplogroup D/E-YAP was significantly over-represented in their prostate cancer patients and haplogroup O-SRY (including the sublineage O-47z; O2b* and O2b1 respectively in their paper) was significantly under-represented. The absence of the haplogroup D/E-YAP from our Korean sample (0%) made it impossible to assess the correlation between this lineage and the cancer cases (Figure 1). However, we could evaluate the protective effect of the O-SRY lineage. In the Korean sample, no protective effect was seen (OR 1.05 (0.58–1.92), p = 0.87; Table 1). These differences could reflect false positive associations in the previous studies, or a genetic susceptibility expressed by Japanese living in a different environment: the patients examined by Paracchini et al. [12], for example, were from the US. However, the effects do not seem to be a general feature of east Asian populations since they are not detected in our additional samples from Korea. It is still desirable to study other populations where the lineages are common.

**Table 2 pone-0000172-t002:**
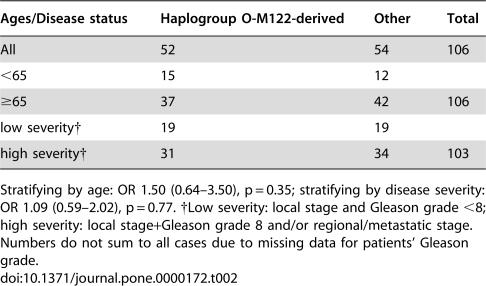
Distribution of the haplogroup O-M122-derived lineages versus all other lineages combined in Korean prostate cancer patients surveyed here

Ages/Disease status	Haplogroup O-M122-derived	Other	Total
All	52	54	106
<65	15	12	
≥65	37	42	106
low severity†	19	19	
high severity†	31	34	103

Stratifying by age: OR 1.50 (0.64–3.50), p = 0.35; stratifying by disease severity: OR 1.09 (0.59–2.02), p = 0.77. †Low severity: local stage and Gleason grade <8; high severity: local stage+Gleason grade 8 and/or regional/metastatic stage. Numbers do not sum to all cases due to missing data for patients' Gleason grade.

Recent surveys from Asia (e.g., Japan, Singapore and Korea) have shown a general trend of a rising incidence of prostate cancer, although the incidence is still lower in Asia than in Western countries [26]. Seem and Cheng [27] noted that the increases in age-adjusted mortality rates per 100,000 person-years, adjusted to the world standard, ranged from 50% in Thailand to 260% in Korea. The changing demography of prostate cancer in Asia may be explained by environmental factors. Many Asian countries may be losing their protective dietary habits and acquiring high-risk ones by adopting westernized lifestyles [27]. Thus, further studies with other diverse samples may be required to evaluate joint actions of genetic background and environmental factors for fuller understanding of the oncogenesis of prostate cancer.

## Methods

### Patients and controls

We analyzed a total of 106 Korean prostate cancer patients, who were recruited for the study from the urology department of the Eulji University School of Medicine in Seoul and Daejeon, Korea. Histological classification of prostate cancer was determined according to the World Health Organization (WHO) recommendation and the Gleason pattern. Prostate cancer tissue specimens from all of the patients were collected from frozen samples. In addition, a total of 110 Korean men who had been diagnosed as free of prostate cancer by the Eulji University hospital in Seoul and Daejeon, Korea were recruited as normal controls. These subjects were selected at random (and therefore likely to be unrelated) from the same geographical area as the cases. This study was approved by the Ethics Committee of Eulji Medical Center of the Eulji University School of Medicine in Seoul, and informed consent was obtained from all participants.

DNAs were prepared from the prostate cancer specimens of patients and whole blood samples of controls according to standard methods [28].

### Genotyping

Fourteen Y-chromosomal binary markers were chosen to genotype all individuals sampled: YAP [29], M7, M9 [30], RPS4Y_711_ [31], SRY_+465_, DXYS5Y [32], P31 [33], M95, M119, M122, M134, M175, M214 [16], LINE1 [34]. All are known to be polymorphic in east Asia. The Y *Alu* insertion (YAP), RPS4Y_711_ (C to T substitution), M9 (C to G substitution), M175 (−5 bp), M95 (C to T substitution), SRY_+465_ (C to T substitution), DXYS5Y (G to C substitution), and LINE1 insertion were typed using the previously described protocol [20].

The M7 (C to G substitution), M134 (−1 bp), M214 (T to C substitution), M119 (A to C substitution), P31 (T to C substitution), and M122 (T to C substitution) markers were amplified using the following primer sets and modifications reported by Hammer et al. [33] and Underhill et al. [16], [30]: M7, 5′-CTTGACCAATGCCTTGCAAA-3′ and 5′-CAGCCTTGTGATCCAATTA-3′; M134, 5′-AATCATCAAACCCAGAAGGG-3′ and 5′-CCTTGTTAGCTAATTTTGAGC-3′; M214, 5′-TGCTGATACAACACACTGGA-3′ and 5′-AGCCATGGAAATGCCACTTCAC-3′; M119, 5′-GTTATGGGTTATTCCAATTCAGC-3′ and 5′-GAATGCTTATGAATTTCCCAGA-3′; P31, 5′-TAAGGCTGCGTGTTCCCTAT-3′ and 5′-ATATCGTGCCATTGCACACC-3′; M122, 5′-CAGCGAATTAGATTTTCTTGC-3′ and 5′-TGGTAAACTCTACTTAGTTGCCTTT-3′. Each PCR reaction was performed in a total volume of 25 µl containing 25 ng of genomic DNA, 10 pM each primer, 0.2 mM dNTPs, 2.0 mM MgCl_2_, 50 mM KCl, 10 mM Tris-HCl (pH 8.3), and 1.5 U Ampli*Taq* DNA polymerase (Perkin-Elmer, Foster, CA, USA). The PCR cycling conditions for the M7 marker used a first denaturation step at 94°C for 5 min, and then 35 cycles at 94°C for 45 sec, 54°C for 45 sec, 72°C for 1 min, and a final extension at 72°C for 3 min. The cycling conditions for the M134 marker used a first denaturation step at 94°C for 5 min, and then 35 cycles at 94°C for 45 sec, 55°C for 45 sec, 72°C for 1 min, and a final extension at 72°C for 3 min. The cycling conditions for the M214 marker used a first denaturation step at 94°C for 5 min, and then 35 cycles at 94°C for 45 sec, 53°C for 45 sec, 72°C for 1 min, and a final extension at 72°C for 3 min. The cycling conditions for M119 were 94°C for 5 min, and then 35 cycles at 94°C for 45 sec, 56°C for 45 sec, 72°C for 45 sec, and a final extension at 72°C for 5 min. P31 was amplified with the PCR conditions of 95°C for 5 min, and then 35 cycles at 94°C for 30 sec, 56°C for 30 sec, 72°C for 45 sec, and a final extension at 72°C for 2 min. The cycling conditions for the M122 marker were 94°C for 5 min, and then 35 cycles at 94°C for 1 min, 54°C for 1 min, 72°C for 1 min, and a final extension at 72°C for 2 min. The PCR products for M122 were digested with *Hsp*92II enzyme (New England Biolabs, Beverly, MA, USA) and fractionated on 2% agarose gel. Mutations of the M7, M119, M134, M214 and P31 markers were detected by a PCR-SSCP method after PCR amplification described by Kutach et al. [35]. The band patterns of their alleles were evaluated on a 10% native PAGE gel run at 10°C in a cold chamber and visualized by silver staining as described elsewhere [36].

Y-chromosomal binary haplogroups for all samples of prostate cancer cases and controls were defined by the analysis of all 14 binary polymorphisms. The nomenclature of the haplogroups followed that of the Y chromosome consortium (YCC) [37].

### Data analyses

Y haplogroup frequencies were calculated by counting from the observed phenotypes. To test for significant population differentiation between the prostate cancer cases and the control groups, we used a Chi squared test and Fisher exact test implemented in the Arlequin package version 2.0 [38]. The significance level of the test was applied with a probability of <0.05 as cutoff point. In addition, a test of proportion and odds ratios (OR) with 95% confidence intervals (CI) were also calculated (http://home.clara.net/sisa/).
